# Speech-language therapists’ views of oral feeding of infants on high-flow oxygen

**DOI:** 10.4102/hsag.v30i0.2809

**Published:** 2025-02-14

**Authors:** Andile Dludla, Tarryn B. Forman, Mikaela K. Lloyd, Savannah O. Moodley, Sarveshvari B. Pillay, Esedra Krüger

**Affiliations:** 1 Department of Speech-Language Pathology and Audiology, Faculty of Humanities, University of Pretoria, Pretoria, South Africa

**Keywords:** high-flow oxygen, oral feeding, nCPAP, HFNC, speech-language therapists, infant, young child, survey

## Abstract

**Background:**

Introduction of oral feeding for young children receiving high-flow oxygen has recently gained interest. With limited literature, there are varied opinions regarding the safety of oral feeding in this population.

**Aim:**

This study describes speech-language therapists’ (SLTs) views on oral feeding for infants receiving high-flow oxygen.

**Setting:**

A South African online survey study.

**Methods:**

A descriptive quantitative survey was distributed electronically via social networking sites. Purposive and snowball sampling were used to recruit expert SLTs. Twenty-one South African SLTs working with paediatric swallowing and feeding, from nine provinces responded. Data were analysed descriptively.

**Results:**

Of 21 responses, only nine were fully complete, indicative of how few South African SLTs work with infants on high-flow oxygen. Current oral feeding practices varied with differences between nasal continuous positive airway pressure (nCPAP) and high-flow nasal cannula (HFNC). Strategies for oral feeding included volume and time limitations, monitoring physiological stability and assessing for aspiration. Thin liquids were most commonly used. Varied opinions, with no protocols or guidelines for introduction of oral feeding of young children on high-flow oxygen, are reported.

**Conclusion:**

SLTs’ practices regarding oral feeding in infants/children receiving high-flow oxygen are variable. Professionals share common approaches to determine feeding readiness and monitor tolerance. Without guidelines and standardised protocols, SLTs are left to make decisions based only on experience. A need exists for further research.

**Contribution:**

There is variability in initiation of oral feeds, highlighting the need for further data to inform uniform protocol and guideline development to enhance SLTs’ decision-making.

## Introduction

Infants and young children in the Paediatric and Neonatal Intensive Care Units (PICU and NICU) often receive high-flow oxygen through Nasal Continuous Positive Airway Pressure (nCPAP) or High-Flow Nasal Cannula (HFNC) support (Canning et al. 2021). This supplemental oxygen provides crucial respiratory support to neonates experiencing respiratory distress and chronic neonatal lung disease, as well as to children with conditions such as bronchiolitis and hypoxaemia (Canning et al. 2021; Moreel & Proesmans 2020). Traditionally, patients on high-flow oxygen have received nutrition only through gastrointestinal (GI) tube feeding or parenteral methods (Canning et al. 2020). However, there is growing interest in the feasibility of oral feeding during high-flow oxygen support because of its potential benefits (Gray et al. 2023). The views of speech-language therapists (SLTs) with this population remain undetermined, warranting further investigation.

Children admitted to the PICU and needing high-flow oxygen often do not want to or cannot feed orally, resulting in nutrition via enteral feeding, most commonly through nasogastric tubes (Morton et al. 2019). These children usually have established feeding skills before PICU admission, so receiving parenteral nutrition does not generally disrupt their feeding skill development (Canning et al. 2020). In contrast, preterm infants in the NICU often receive ventilation and high-flow oxygen because of immature lung development and have not yet established oral feeding. Sick newborns such as those with hypoxic-ischaemic encephalopathy are also at risk of feeding difficulties and require additional support to establish oral feeding (Krüger et al. 2017). Promoting oral feeding skill development in these patients can help decrease the length of hospitalisation if performed safely by a qualified and trained professional with the relevant expertise (Kamity, Kapavarapu & Chandel 2021).

Research on the appropriateness of high-flow oxygen use for orally fed infants is limited (Dumpa et al. 2020; Murphy, Harrison & Harding 2018). Some evidence suggests that delaying oral feeding for infants on nCPAP does not necessarily result in feeding difficulties (Dumpa et al. 2020). However, delayed oral feeding can negatively impact an infant’s or young child’s overall development because of prolonged NICU or PICU stays (Jadcherla & Bhandari 2017; Robinson, Heng & Fucile 2022). Early identification of feeding readiness and the introduction of oral feeding are known to shorten hospitalisation, reduce familial stress and lessen the economic burdens associated with extended hospital stays (Crenshaw 2018; Leder et al. 2016).

The safety of oral feeding during HFNC and nCPAP use is uncertain, particularly concerning airway pressure during feeding therapy (Canning et al. 2020). The risk of laryngeal penetration and aspiration, potentially leading to respiratory infection in patients with bronchopulmonary dysplasia (BPD), discourages aggressive oral feeding interventions (Jadcherla & Bhandari 2017). Despite these concerns, there are benefits to oral feeding during HFNC. Shetty et al. (2016) found that orally fed infants on nCPAP reached full oral feeding approximately 17 days faster than those fed via non-oral methods. Therefore, infants on non-invasive respiratory support may benefit from focussed and individualised oral motor feeding strategies to reach feeding milestones (Jadcherla & Bhandari 2017). In the PICU, oral feeding can benefit young children with respiratory illnesses such as pneumonia and bronchiolitis by providing optimal nutrition, reducing hospitalisation length, and increasing comfort for infants and families (Canning et al. 2021). Small amounts of specific consistencies introduced carefully within a consultation with a feeding team may be possible (Hoosain et al. 2024).

Speech-language therapists are integral decision-makers in the feeding team; the role of the SLT in managing oropharyngeal dysphagia in infants and children with pulmonary compromise is well-established (American Speech-Language and Hearing Association [ASHA] 2022). While there are advantages to oral feeding, health professionals, including SLTs, express concerns about oral feeding safety for infants receiving high-flow oxygen. The consensus on the safety and risks of oral feeding varies between professionals and hospitals (Canning et al. 2020). The relationship between feeding and aspiration risk is critical, and there is no definitive evidence to support oral feeding and swallowing on high-flow oxygen (Dumpa et al. 2020). It is not the use of high-flow oxygen that should preclude oral feeds but rather patient-specific factors related to oral readiness and underlying medical conditions that increase aspiration risk (Hoosain et al. 2024). High-flow oxygen therapy in itself should not be the reason to withhold oral feeding, but without data about safety from large studies, SLTs have to proceed with caution (Rice & Lefton-Greif 2022).

While some tertiary hospitals or specialist institutions in South Africa may have feeding protocols indicating that patients should not be fed on high-flow oxygen because of the risk of aspiration, there are no locally published guidelines on oral feeding introduction for infants on high-flow oxygen in the public or the private healthcare sector (Hoosain et al. 2024). However, international guidelines, such as those developed by Conway et al. (2021), provide specific criteria for HFNC management, including flow rates and inclusion or exclusion criteria based on respiratory status, age, medical history and Bronchiolitis Scoring System scores. Patients must also meet specific respiratory rate criteria and receive clearance from the clinical team before oral feeding can commence (Conway et al. 2021).

Professionals in the NICU and PICU may have differing opinions on the safety of oral feeding on high-flow oxygen, including suitable oxygen concentration or pressure for oral feeding and case management differences (Canning et al. 2021). By determining the perspectives of SLTs, who are considered the paediatric oral swallowing and feeding experts (ASHA 2022), this study aims to understand professional views on a global issue within a South African context. The following research question was posed: What are the views of SLTs regarding oral feeding with infants and young children receiving high-flow oxygen?

## Research methods and design

### Aim

To describe the views of SLTs regarding oral feeding with infants and young children receiving high-flow oxygen.

### Study design

A cross-sectional electronic survey design rendering predominantly quantitative data was used. A previously published survey was adapted for this study (Canning et al. 2020).

### Study population and sampling strategy

The study used purposive and snowball sampling allowing participants to share the survey with other potential participants in their networks (Brink, Van Der Walt & Van Rensburg 2018). The study was open to South African SLTs who met the following inclusion criteria: Participants had to have at least 3 years’ experience with feeding intervention and high-flow oxygen in the NICU and/or PICU (Rowland & Adefuye 2022) and be registered with the Health Professions Council of South Africa at the time of the study. Participants were recruited via an advertisement and electronic survey link distributed through the South African Speech-, Language- and Hearing Association (SASLHA) newsletter and social networking sites. In total, 43 responses were obtained, while only 21 responses were suitable for inclusion. This was because of respondents not fulfilling the required criteria of a minimum of 3 years’ experience, or not consenting to the study. The sample size was limited to those who volunteered to participate in the study. Given the specialist nature of the sample and the limited number of SLTs who work in paediatric dysphagia management in South Africa, it was expected that the sample size would be limited. Therefore, no formal power analysis was conducted, and all potential participants were included. Characteristics of participants are reflected in Table 1.

**TABLE 1:** Participant characteristics (*N* = 21).

Characteristics	*n*	%
**Province (*n* = 21)**		
Eastern Cape	2	9.5
Free State	0	0
Gauteng	12	57.0
KwaZulu-Natal	2	9.5
Limpopo	1	4.8
Mpumalanga	0	0
Northern Cape	0	0
North West	0	0
Western Cape	4	19.0
**Healthcare sector (*n* = 21)**		
Private	8	38.1
Public	13	61.9
**Wards worked in**		
Neonatal Intensive Care Unit (NICU)	16	35.6
Paediatric Intensive Care Unit (PICU)	9	20.0
Kangaroo Mother Care (KMC)	14	31.1
Other	6	13.3
**High-flow oxygen use in units**		
nCPAP (*n* = 20)	17	85.0
HFNC (*n* = 10)	9	90.0

nCPAP, nasal cannula positive airway pressure; HFNC, high-flow nasal cannula.

The majority of participants resided in Gauteng (*n* = 12; 57%), and worked mainly in public healthcare (*n* = 13; 61.9%) and the NICU (*n* = 16; 35.6%). Out of a total of 11 participants, 10 (90.91%) indicated that HFNC is used in their ward(s). Out of a total of 20 participants, 17 (85.00%) indicated that nCPAP is used in their ward(s).

### Materials

The published survey by Canning et al. (2020), who studied oral feeding during high-flow oxygen in New Zealand and Australia among intensive care workers, was adapted for the purpose of the study (see Supplementary material). The electronic survey consisted of mostly closed-ended questions that were modified to better serve South African participants, with the inclusion of specified options to reduce ambiguity. The survey was pretested prior to publishing the link. Based on the feedback received during the pretest, editorial adjustments were made to specific survey questions. These adjustments involved adapting the setting to South Africa, clarifying question wording, revising a few response options and ensuring the overall coherence of the survey. The survey, made up of 41 questions, generated quantitative data and consisted of yes or no questions, multiple choice and simple one-word answers. An open-ended question was included at the end of the survey, whereby participants were asked to provide the differing opinions of staff members within the unit, regarding oral feeding of infants and children receiving high-flow oxygen support to add qualitative data.

### Data collection

An infographic with an active Qualtrics survey link was distributed via social media platforms, the SASLHA newsletter and researchers’ personal networks. The survey was launched on 25 April 2023 and was available for 3 months, with monthly reminders on social media pages, to ensure that all potential participants could access the survey within the time frame specified for data collection. Data were collected in accordance with the *Protection of Personal Information Act* (POPIA 2022); no identifying information, contact information or IP addresses were collected.

### Data analysis

The data were downloaded and depicted graphically using MS Excel. Descriptive statistics were used to summarise and organise the data (Kaur, Stoltzfus & Yellapu 2018).

### Reliability and validity

Utilising a previously published survey allows researchers to build on previous findings, contributing to the cumulative body of knowledge on a particular topic thus showing content validity (Brink et al. 2018). Purposive sampling additionally increased the range of specific information obtained through targeting only SLTs who had experience with the use of high-flow oxygen while feeding infants and children, thus increasing the reliability and validity of data (Brink et al. 2018). A pretest of the survey was conducted with a small group of undergraduate Speech-Language Pathology students and staff members. This was carried out to ensure the clarity of the questions, the proper functioning of the Qualtrics survey link, the accurate capturing of data and to verify the time taken to complete the survey.

### Ethical considerations

Institutional ethical clearance was obtained at the University of Pretoria from the Department of Speech-Language Pathology and Audiology Research Committee (reference no.: SLPA2023/02) prior to data collection. Participants’ identities were kept anonymous to researchers, facilitated by the online survey format. Electronic informed consent was requested at the beginning of the survey. Participants were informed of their right not to participate and that they could not withdraw their information after submitting the survey. Participants’ answers were saved automatically; if they stopped completing the survey, this was processed as missing data and reported as such. The data collection process and the safekeeping of data were explained at the beginning of the survey. Participants indicated via a checkbox that they had read and understood the consent (after being provided with an HTML link to access the information leaflet explaining the study and their rights as participants), thus choosing to continue with the survey.

## Results

A total of 43 responses were obtained, and 22 of these responses were excluded from analysis because of not meeting the predefined inclusion criteria, leaving 21 eligible surveys for analysis. Out of these 21 responses, only nine were fully completed. Missing data are indicated throughout.

### Defining high-flow oxygen

Nine participants reported on the definition of ‘high-flow’ oxygen support. High-flow nasal cannula was defined as > 2 L/min by one (11.0%); >1 L/min by three (33.0%), L/kg by 11.0% (*n* = 1) and 44.4% (*n* = 4) of the nine answering the question, were uncertain about the specific definition of HFNC.

### Frequency of oral feeding on nasal cannula positive airway pressure and high-flow nasal cannula

When assessing the frequency of oral feeding while infants and children were on nCPAP (*n* = 15) or HFNC (*n* = 9), findings reveal that for nCPAP, five out of 15 (33.3%) participants indicated infants and children are *rarely* or *never* fed orally, while four out of 15 (26.7%) reported that oral feeding *sometimes* occurs in their units. One out of 15 (6.7%) stated that oral feeding is *often* conducted when on nCPAP. Four participants (45.0%) reported that infants and children are *sometimes* fed orally on HFNC. Three participants (33.0%) mentioned daily oral feeding, while one participant (11.1%) stated that infants and children are *never* fed orally, and one participant (11.1%) reported *rarely* providing oral feeds on HFNC.

### No oral feeding on nasal cannula positive airway pressure or high-flow nasal cannula

Participants were asked about factors influencing decisions not to administer oral feeds to infants and children on nCPAP and/or HFNC. Five out of 15 (33.3%) reported that they never administered oral feeds on nCPAP, although they did not specify the rationale for this decision. Only one participant (7.0%) indicated that infants and children receiving HFNC were never provided with oral feeding because of the aspiration risk associated with this practice. Methods of oral feeding on nCPAP and HFNC are presented in Table 2. Only nine participants completed this question.

**TABLE 2:** Oral feeding methods used for infants or children receiving nasal cannula positive airway pressure (*n* = 9) compared to high-flow nasal cannula (*n* = 8).

Oral feeding method	HFNC *n* = 8		nCPAP *n* = 9	
	*n*	%	*n*	%
Direct breastfeeding	4	50.0	6	66.6
Bottle feeding	5	62.5	3	33.3
Infant cup	3	37.5	5	55.5
Syringe	5	62.5	3	33.3
Cup (sipper/straw/open cup)	2	25.0	1	11.1
Solids	1	12.5	1	11.1
Other	0	0	0	0

Note: Participants could select more than one option.

nCPAP, nasal cannula positive airway pressure; HFNC, high-flow nasal cannula.

Participants’ choice of strategies for oral feeding on HFNC varied based on factors such as the underlying condition, therapy duration, child’s age and aspiration risk (Table 3). Only nine participants completed this question.

**TABLE 3:** Strategies employed by speech-language therapists to support oral feeding on nasal cannula positive airway pressure (*n* = 9) and high-flow nasal cannula (*n* = 8).

Strategies	nCPAP *n* = 9		HFNC *n* = 8	
	*n*	%	*n*	%
Volume limited feeds	4	44.4	4	50.0
Time-limited feeds	6	66.7	6	75.0
Monitoring of physiological stability	7	77.8	6	75.0
Respiratory support is reduced	7	77.8	5	62.5
Specific criteria for respiratory stability are required (e.g. respiratory rate)	4	44.4	-	-
Monitoring for clinical signs of aspiration	7	77.8	-	-
Positioning modifications	4	44.4	-	-
Therapeutic tastes	2	22.2	-	-
Specific feeding equipment (e.g. type of teat)	2	22.2	-	-
None	1	11.1	-	-
Other	0	0	1	12.5

Note: Participants could select more than one option.

nCPAP, nasal cannula positive airway pressure; HFNC, high-flow nasal cannula.

### Specific criteria for respiratory stability required (e.g. respiratory rate) for an infant or child on high-flow nasal cannula to be fed orally

Eight participants indicated specific criteria used within their unit to ensure respiratory stability, most frequently reporting that positioning modification (*n* = 7; 87.5%) is utilised, followed by monitoring for clinical signs of aspiration (*n* = 5; 62.5%) and using specific feeding equipment (e.g. differing bottle teats) (*n* = 4; 50.0%). Supervising the oxygen flow rate by litres per minute (L/min) or litres per kilogram (L/kg) (*n* = 4; 50.0%) was additionally a strategy used. Less frequently, therapeutic tastes (*n* = 3; 37.5%) were included as a criterion for respiratory stability.

### Food textures or fluid consistencies provided to infants or children receiving nasal cannula positive airway pressure and high-flow nasal cannula

Participants described consistencies provided to infants and young children receiving nCPAP and HFNC from a specified list described according to the International Dysphagia Diet Standardisation Initiative [IDDSI] (Table 4). Thin liquid was most frequently provided for both patients on nCPAP (*n* = 8; 42.1%) and HFNC (*n* = 7; 38.9%). Nineteen participants answered this question.

**TABLE 4:** Food textures and fluid consistencies provided on nasal cannula positive airway pressure (*n* = 19) and high-flow nasal cannula (*n* = 18) based on the international dysphagia diet standardisation initiative.

Fluid consistency or food texture	nCPAP *n* = 19		HFNC *n* = 18	
	*n*	%	*n*	%
Thin fluids (water, breastmilk, formula and fruit juice)	8	42.1	7	38.9
Thickened fluids (thickened milk, nectar-thickened juice and honey-thickened fluids)	2	10.5	3	16.7
Purees (pureed fruits, vegetables and meats)	4	21.1	3	16.7
Lumpy mashed foods (mashed potatoes with lumps and mashed bananas with small pieces)	2	10.5	2	11.1
Minced and moist foods (minced chicken with gravy and minced vegetables with sauce)	2	10.5	1	5.6
Chewable foods (soft foods like bananas, well-cooked pasta and tender cooked meats like chicken or fish)	1	5.3	2	11.1

Note: Participants could select more than one option.

nCPAP, nasal cannula positive airway pressure; HFNC, high-flow nasal cannula.

### Restrictions to food textures or fluid consistencies

Restrictions to food textures or fluid consistencies were more frequently in place for patients receiving HFNC (*n* = 5; 62.5%), however, did not describe the type of restrictions applicable. Less than half of the participants (*n* = 3; 37.5%) indicated there were no restrictions in place for this population. Contrastingly, in the answers of those who selected nCPAP (*n* = 9), it was indicated that there were more frequently no restrictions to fluid consistencies or food textures (*n* = 5; 55.6%). Participants who selected restrictions (*n* = 4; 44.4%) for infants and children receiving nCPAP described that these patients were limited to breast milk, donor breast milk, and supplemented formula milk. If on liquid diets and purees, they may also receive those. Solid foods were described as rare. Participants indicated that these restrictions depended on the patient’s aspiration risk, age and underlying condition.

### Feeding management

#### Commencement or recommencement of oral feeding

Participants were asked whether the commencement or recommencement of oral feeding for infants and children is a team decision within their clinical context (*n* = 9). Eight (88.9%) indicated that it is a team decision. Additionally, when asked to identify primary decision-makers, the majority of participants acknowledged the significant roles of SLTs (42.1%) and medical doctors (26.3%), with nursing staff (15.8%) and occupational therapists (5.3%) also playing a key role. One participant (11.1%) indicated the involvement of dieticians, while only one participant (11.1%) indicated it is always a team decision.

#### Criteria or tools used to assess infant or child readiness for oral feeding

The majority of the sample of nine participants emphasised the importance of observing feeding readiness cues (*n* = 6; 66.7%), highlighting the importance thereof in assessment (Table 5).

**TABLE 5:** Criteria used to assess infant or child readiness for oral feeding (*N* = 9).

Criteria	*n*	%
Age	4	44.4
Weight	6	66.6
Observation of feeding readiness cues	6	66.6
Cardiorespiratory stability	5	55.5
Resolution/improvement of current illness	2	22.2
Workplace guidelines	2	22.2
No longer on nCPAP	5	55.5
No longer on HFNC	4	44.4
Specific flow rate (L/min, L/kg, cmH2O)	2	22.2
Oral feeding readiness tool: Feeding diary	3	33.3
Other	1	11.1

Note: Participants could select more than one option.

nCPAP, nasal cannula positive airway pressure; HFNC, high-flow nasal cannula.

#### Written policies or guidelines

Only two participants out of nine (22.3%) indicated that their unit has a written policy or guideline that includes feeding recommendations for infants or children receiving non-invasive respiratory support.

#### Feeding specialist services and assessment tools

All participants (*n* = 9; 100%) who answered the question indicated that specialised feeding assessment and intervention services were provided by SLTs only. Few participants (*n* = 2; 22.3%) provided services daily, while another provided services three times (*n* = 1; 11.1%) or twice (*n* = 1; 11.1%) per week. Several participants (*n* = 7; 77.7%) use formal or informal feeding evaluation tools to assess oral sensorimotor, feeding and swallowing function or competence while few participants (*n* = 2; 22.2%) do not. The majority of participants (*n* = 7; 77.7%) did not have access to instrumental evaluations or monitoring of swallowing to assess the swallowing safety of patients. Those who have access (*n* = 3; 33.3%) used Video-Fluoroscopic Swallow Study (*n* = 2; 22.2%), Modified Barium Swallow (*n* = 2; 22.2%), Fiberoptic Endoscopic Evaluation of Swallowing (FEES) (*n* = 1; 11.1%) and pulse oximetry (*n* = 1; 11.1%).

#### Participant self-rating of knowledge, confidence and experience

While most participants indicated that they agree that they are knowledgeable (*n* = 7; 77.8%), in treating infants or young children receiving high-flow oxygen, fewer participants indicated feeling confident (*n* = 6; 66.6%) and experienced (*n* = 5; 55.5%).

### Reliability and validity

By utilising a published survey, researchers can build on previous findings, contributing to the cumulative body of knowledge on a particular topic and demonstrating content validity (Brink et al. 2018). Purposive sampling further enhanced the range of specific information gathered by targeting only SLTs with experience in using high-flow oxygen while feeding infants and children, thus increasing the reliability and validity of the data obtained (Brink et al. 2018). The automatic process of compiling answers in an online form minimised data-capturing errors by the researchers, further enhancing the reliability of the data (Brink et al. 2018).

## Discussion

This study aimed to describe the views of South African SLTs regarding oral feeding of infants and children on high-flow oxygen. Despite a small sample size, the study yielded clinically valuable findings based on the perspectives of skilled clinicians. Practices varied regarding the frequency of oral feeds, different consistencies of feeds, nutritional support methods and criteria used to evaluate oral feeding safety for patients receiving high-flow oxygen. This variation likely reflects the current uncertainty regarding the safety of oral feeding while on high-flow oxygen and the wide range of populations and settings in which the SLTs in this study work. Many participants indicated that they work in multiple settings or units, with the majority working in the NICU and Kangaroo Mother Care (KMC) units. There was no clear distinction found between the views of SLTs in the private versus public sector in this study. Most participants reported feeling knowledgeable in working with infants and children on high-flow oxygen, indicating a general competence in managing paediatric swallowing and feeding difficulties. Interestingly, fewer participants perceived themselves as confident and experienced.

The definition of high-flow oxygen varied among participants in this study, with many indicating they were unsure of the definition in their own units, which is concerning. The importance of defining high-flow oxygen and its subsequent impact on oral feeds cannot be understated, as flow rates impact infants’ or children’s feeding because of their size or weight (Canning et al. 2021). The high-flow nasal cannula is best described as a well-adjusted flow rate according to the patient’s specific variables, such as litres per minute per kilogram (Canning et al. 2021). Speech-language therapists and other healthcare professionals require knowledge of the appropriate flow rate, individualised per patient, to guide subsequent decisions surrounding oral feeds. High-flow nasal cannula and nCPAP have been shown to have similar success rates in newborns at an oxygen flow rate of less than 2 litres per minute (Luo et al. 2022), and the practices of SLTs mirror this, with most participants indicating a threshold under this limit.

Those who indicated that they rarely orally fed infants or children receiving nCPAP work with a wide range of ages (preterm neonates to school-aged children), which likely explains the variability in responses related to preferable methods of oral feeding, from breastfeeding to providing solids. Breastfeeding and infant cups were most frequently the oral feeding methods of choice, likely because of the similar movement of the lingual and masseter muscles (Franca et al. 2014). Participants indicated a preference for non-oral methods of nutrition for their patients receiving both nCPAP and HFNC support, with most opting for non-oral feeding via both methods of respiratory support respectively. It appears that the majority of SLTs in this study do not consider patients receiving these methods of respiratory support as suitable or stable enough to safely receive their feeds orally. Therefore, the preference for such non-oral feeding methods among participants should be further explored, with emphasis on whether the decision is informed by SLTs or other medical professionals.

Variation was found in the frequency of oral feeds when an infant or child received nCPAP compared to HFNC. Those on nCPAP were fed orally less frequently than those receiving HFNC, likely because of participants’ awareness of the risk of aspiration as reported in open-ended questions. Recent research highlights the lack of strong data supporting oral feeding practices for infants and children receiving non-invasive ventilation, emphasising the variability among studies and the lack of an evidence-based standard of care (Barnes, Herbert & Bonilha 2023).

In contrast, infants and children on HFNC were predominantly orally fed through bottle feeding and syringes. This might be because older infants (Luo et al. 2022) had pre-established feeding skills or were familiar with bottle feeding, which could be more efficient and promote better weight gain compared to breast- and cup-feeding in infants with possible feeding difficulties (Alinezhad Shebilouysofla et al. 2022).

The management of dysphagia by an SLT involves assessing swallowing function across a variety of food textures and drink thicknesses, standardised by the IDDSI (Cichero et al. 2017). Drink thicknesses provided to infants or children receiving either nCPAP or HFNC were predominantly thin fluids and/or purees, followed by thickened fluids. Despite this, it is known that consistencies of thicker viscosity enhance swallowing by allowing for the creation of a more cohesive bolus, increasing oropharyngeal transit time, ultimately normalising swallowing patterns during respiration, acting as a potential strategy to reduce the risk of aspiration (Brooks et al. 2022; Wolter et al. 2018).

While all participants in this study described that specialised feeding assessment and intervention services were provided by SLTs in their units, many participants did not have access to instrumental assessment methods. Instrumental assessment is required to definitively diagnose the type and severity of dysphagia, as a clinical evaluation merely detects the possibility or presence thereof (Kamity et al. 2020). In low- to middle-income countries, such as South Africa, access to instrumental assessment is scarce. Several participants indicated using formal or informal feeding evaluation tools to assess oral sensorimotor, feeding and swallowing functioning or competence, including feeding diaries as an oral feeding readiness tool. Tools such as the locally developed Neonatal Feeding Assessment Scale (NFAS) or the Early Feeding Skills Assessment Tool (EFS) are valid and reliable tools to assess oral feeding readiness and may inform hospital-specific oral feeding readiness tools or feeding diaries (Aykanat Girgin et al. 2021; Viviers et al. 2017). Clinical evaluation of physiological symptoms was therefore primarily relied upon according to the results of this study. Clinical assessment provides valuable information regarding physiological symptoms during swallowing, allowing for diagnostic recommendations to be made when combined with clinical expertise (Coutts & Pillay 2021). Behavioural cues, such as hiccuping, cyanosis and wet and/or gurgly breathing (Bowman et al. 2020) were primarily used to indicate poor tolerance of oral feeds. Other measures, including decreases in physiological stability, clinical signs of aspiration or laryngeal penetration, such as coughing, and changes in state and organisation of sucking, swallowing and breathing, were equally used to indicate tolerance to oral feeds. The strategy most frequently employed to monitor oral feeding tolerance for all patients on non-invasive ventilation was monitoring physiological stability. Monitoring physiological stability to determine the tolerance of oral feeds includes factors such as monitoring heart rate, respiration rate and oxygen saturation (Astuti, Rustina & Wanda 2022; Lund 2021). It may also be defined as the stability of the coordination of the suck, swallow and breathe pattern if developmentally appropriate (Astuti et al. 2022).

Clinical experience and expertise were the most variable responses in the self-rating question of this study, indicating a lack of perceived expertise or experience in this field. Expertise regarding the quadruple burden of disease and the effect on swallowing physiology is important for SLTs practising in South Africa because of the complexity of medical conditions that these professionals encounter (Stone et al. 2020). South African SLTs would greatly benefit from standardised guidelines on oral feeding of children and infants while on high-flow oxygen to support inexperienced clinicians in making ethical decisions that benefit clients and families. The lack of published policies or guidelines that include swallowing and feeding recommendations for infants or children receiving non-invasive respiratory support indicated by the majority of participants highlights a caveat regarding standardised practices both globally and locally. This finding echoes the conclusion of Canning et al. (2020), which reported that the majority of units in Australia and New Zealand had no written guidelines or policies outlining feeding recommendations for infants or children on high-flow oxygen support. Clinicians such as SLTs and other stakeholders in hospital settings are thus urged to develop guidelines for SLTs to guide decision-making.

Increasing evidence describes that the oral feeding of infants or children receiving HFNC and nCPAP is safe (Conway et al. 2021), where restricting oral feeds is linked to weight loss and longer hospitalisation periods (Shadman et al. 2019). The importance of a team-based approach to feeding decisions is an implication of this study. Inter-professional collaboration in feeding decisions is described as being optimal for the paediatric population (Canning et al. 2021; Coutts & Pillay 2021; Shadman et al. 2019). However, the importance of further research on the introduction of oral feeding in infants and young children on non-invasive oxygen support is emphasised. According to Canning et al. (2021), sufficient or conclusive evidence as to whether oral feeding of infants or children on high-flow oxygen support facilitates the transition to full oral feeding is not yet available. Continued research efforts need to specify the impact of different types and flow rates of high-flow oxygen on the mechanical properties of swallowing through instrumental assessment (Barnes et al. 2023). Children with bronchiolitis on HFNC therapy tolerated oral feeding in one single-centre study (Gray et al. 2023). Another small South African study found partial oral feeding of specific consistencies to be viable when patients’ cases are evaluated individually to determine readiness for oral feeds (Hoosain et al. 2024); however, further research efforts are necessary.

The findings in this study indicate that SLTs will most probably have to assist with decisions about oral feeding in infants and children on high-flow oxygen, suggesting a need for adequate education and support of SLTs. The findings are from a group of SLTs who regularly work with infants and children who have feeding difficulties, providing valuable direction for future research. The variability in participants’ answers indicates the importance of guidelines for SLTs and relevant healthcare professionals on team-based feeding decisions.

## Conclusion

The views of South African SLTs regarding the oral feeding of infants and children receiving HFNC and nCPAP, while aligned with the scope of practice for SLTs, reveal differing perspectives. Responses to the initiation of oral feeds for this population varied among the sample of experienced SLTs, highlighting a lack of clarity among professionals and the limited availability of protocols and guidelines to enhance clinical decision-making. Speech-language therapists and medical professionals are urged to collaborate inter-professionally on feeding decisions for this vulnerable population. Although the study is limited by a small sample size, the findings offer valuable insights into local SLTs’ perspectives on an important global issue. Further research exploring the perspectives of other stakeholders on feeding practices for this group of infants and young children in South Africa is warranted.
